# Stimulated Emission up to 2.75 µm from HgCdTe/CdHgTe QW Structure at Room Temperature

**DOI:** 10.3390/nano12152599

**Published:** 2022-07-28

**Authors:** Vladimir V. Utochkin, Konstantin E. Kudryavtsev, Alexander A. Dubinov, Mikhail A. Fadeev, Vladimir V. Rumyantsev, Anna A. Razova, Egor V. Andronov, Vladimir Ya. Aleshkin, Vladimir I. Gavrilenko, Nikolay N. Mikhailov, Sergey A. Dvoretsky, Frederic Teppe, Sergey V. Morozov

**Affiliations:** 1 Institute for Physics of Microstructures of RAS, 603950 Nizhny Novgorod, Russia; konstantin@ipmras.ru (K.E.K.); sanya@ipmras.ru (A.A.D.); fadeev@ipmras.ru (M.A.F.); rumyantsev@ipmras.ru (V.V.R.); annara@ipmras.ru (A.A.R.); andronov@ipmras.ru (E.V.A.); aleshkin@ipmras.ru (V.Y.A.); gavr@ipmras.ru (V.I.G.); more@ipmras.ru (S.V.M.); 2 Faculty of Radiophysics, Lobachevsky State University, 603950 Nizhny Novgorod, Russia; 3 Institute of Radio Electronics and Information Technologies, Nizhny Novgorod State Technical University n.a. R.E. Alekseev, 603950 Nizhny Novgorod, Russia; 4 Advanced School of General and Applied Physics, Lobachevsky State University, 603950 Nizhny Novgorod, Russia; 5 Institute of Semiconductor Physics, Siberian Branch of RAS, 630090 Novosibirsk, Russia; mikhailov@isp.nsc.ru (N.N.M.); dvor@isp.nsc.ru (S.A.D.); 6 Laboratoire Charles Coulomb, UMR 5221, CNRS-University of Montpellier, 34095 Montpellier, France; frederic.teppe@umontpellier.fr

**Keywords:** HgCdTe, MCT, quantum well, stimulated emission, room temperature, mid-IR, carrier heating

## Abstract

Heterostructures with thin Hg(Cd)Te/CdHgTe quantum wells (QWs) are attractive for the development of mid-infrared interband lasers. Of particular interest are room-temperature operating emitters for the short-wavelength infrared range (SWIR, typically defined as 1.7–3 μm). In this work, we report on the observation of stimulated emission (SE) in the 2.65–2.75 µm wavelength range at room temperature in an optically pumped HgCdTe QW laser heterostructure. We study a series of three samples with lengths ranging from 2.5 to 7 mm and discuss the effects related to the non-uniformity of the excitation beam profile. SE threshold intensity and the magnitude of pump-induced carrier heating are found to be effectively dependent on the chip size, which should be accounted for in possible designs of HgCdTe-based optical converters. We also pay attention to the problem of active medium engineering in order to push the SE wavelength towards the 3–5 µm atmospheric window and to lower the SE threshold.

## 1. Introduction

Developing compact, low-cost mid-infrared (mid-IR) lasers is crucial for many applications such as environmental monitoring, detection of carbohydrate derivatives, and medicine. The short-wavelength part of the IR range contains many absorption lines of gaseous pollutants and industrial and greenhouse gases, such as CO, CO_2_, CH_4_, nitrogen oxides, H_2_S, H_2_O, SO_2_, and many others. Compact and efficient selective sensors for one or several substances can be realized by a combination of a tunable narrowband emitter and a nonselective detector, the technique known in literature as tunable laser absorption spectroscopy (TLAS). While the technology of mid-IR detectors is well developed, and there are many commercially available InGaAs-, InAs-, and HgCdTe-based detectors, the technology of low-cost tunable narrowband emitters is underdeveloped. Though there are many spectroscopic lines of various substances in the whole mid-IR range, researchers focus on spectral ranges where atmospheric absorption is minimal, so-called atmospheric windows (0.8–2.5, 3–5, and 8–14 μm), because these ranges allow more accurate measurements by scanning larger volumes of air. Atmospheric absorption in the 2.5–3 µm range is determined predominantly by H_2_O, CO, and CO_2_, so emitters in this range can be used for the sensing of the aforementioned gases [1,2,3]. As for water, biological tissues usually contain large amounts of it, and its strong absorption near 3 µm leads to a very low penetration depth in tissues, which finds applications in skin treatment, laser surgery, dentistry, and other medicine. Therefore, it is important to develop tunable semiconductor lasers that work at room temperature in the short-wavelength, mid-IR range from 2.5 to 5 µm.

Currently, experimentally demonstrated TLAS systems use interband cascade lasers based on III–V materials [4]. Interband cascade lasers provide continuous-wave emission at room temperature with up to 250 mW of output power [5]. At the same time, mass-production of cascade devices is still challenging due to complicated designs and high manufacturing costs. Contrary to this, type-I QW diode sources are easier to manufacture, and lasing with wavelengths above 2.5 μm was realized in semiconductor systems based on III–V (GaSb, InAs), IV–VI (PbS, PbSe), and II–VI ternary/quaternary alloys [6]. Currently, the longest wavelength of room-temperature emission achieved in III–V type-I QW diodes is equal to 3.5 μm [7]. Still, most of these material systems suffer from poor growth technology, which affects the durability and reproducibility of the sources.

In this regard, the sources based on mercury cadmium telluride (MCT, HgCdTe) are very compelling. HgCdTe technology is well developed and is used for the fabrication of IR detectors [8]; it is also considered a promising material for fabrication of mid-IR sources. Interband recombination and lasing in HgCdTe have been investigated since the mid-1960s [9]. However, room-temperature lasing was only achieved in the short-wavelength part of the mid-IR range (2.2 μm wavelength [10]), and, at longer wavelengths, the critical temperature *T*_max_ at which stimulated emission (SE) can be observed decreases rapidly; *T*_max_ was ~190 K for 2.6 μm lasing [11], while a 5.3 μm laser operated at temperature was as low as 45 K [12]. It is well established that the observed strong thermal quenching of interband lasing from narrow gap materials is related to the activation of non-radiative AR processes [12,13,14]. The AR rate increases with temperature and emission wavelength, which prevents room-temperature operation of the interband sources above a certain wavelength.

AR is a three-particle process in which an electron–hole pair recombines and transfers energy to the third carrier [15]; threshold “bulk-like” eeh AR involves two electrons from the lowest conductive subband and a hole from the highest valence subband without transitions to higher subbands. During the “bulk-like” Auger process, the total kinetic energy of the initial system has to exceed a certain threshold value to comply with energy and momentum conservation principles [16] (p. 191). It was demonstrated that implementation of thin Hg(Cd)Te/CdHgTe QWs with residual Cd in the QW material kept as low as possible within growth technique limitations could greatly suppress “bulk-like” AR [17]. Having implemented such thin QWs, we observed 3.8 µm SE at 240 K [18], 2.8 µm SE at 256 K [19], and 2.45 µm SE at room temperature [20] in “as-grown” planar structures, while the sample with fabricated microdisk resonators provided 4.1 µm SE at 260 K [21].

In this work, we modified the sample design from [20] by realizing slightly lower Cd content in the QWs and barrier layers in order to push the room-temperature SE wavelength further into the infrared, towards the 3–5 µm atmospheric window.

## 2. Materials and Methods

**Figure 1:**
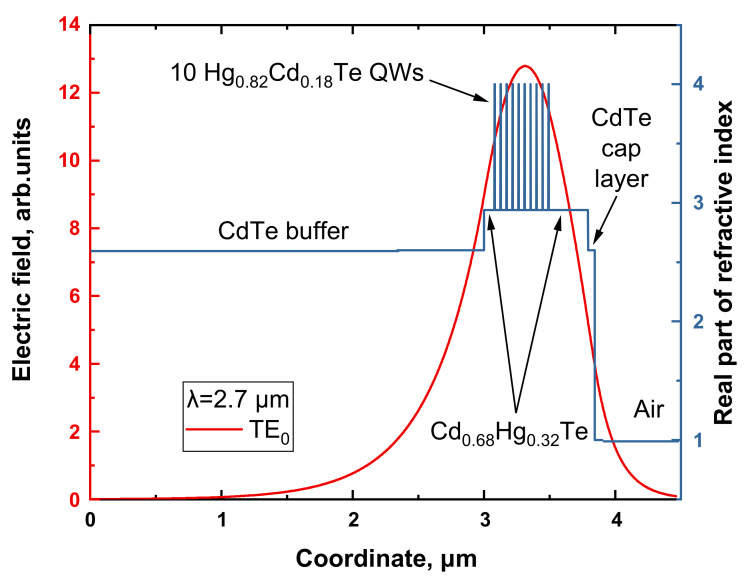
Distribution of real part of refractive index and TE_0_ mode localization for the structure under study at the wavelength λ = 2.7 µm.

The structure studied was MBE grown on semi-insulating GaAs (013) substrate with ZnTe (50 nm thick) and CdTe (10 µm thick) buffers with in situ ellipsometric control of layer thickness and Cd content (see Figure 1). Typical growth parameters and the discussion of MBE process for growth of multiple HgCdTe QWs can be found in [22]. The structure contained 10 Hg_0.82_Cd_0.18_Te/Cd_0.68_Hg_0.32_Te QWs, each 2.7 nm thick in its active area, and was designed to effectively confine guided mode near the QW array, for which the QWs were incorporated into a 800 nm Cd_0.68_Hg_0.32_Te waveguide layer with approximately 900 meV bandgap.

For PL and SE measurements, we chipped a series of three samples with lengths ranging from 2.5 mm to 7 mm (hereafter “2.5 mm”, “4 mm”, and “7 mm”) from the grown structure. For the initial sample characterization via spontaneous PL studies, a continuous-wave (CW) diode laser operating at 808 nm was used as an excitation source; pump density was about 10 W/cm^2^. For SE spectra measurements, a near-IR optical parametric oscillator (OPO) was used, emitting an approximately 2 µm wavelength with an output energy of ~10 mJ in a 10 ns pulse and a repetition rate of 10 Hz. Pump intensity was attenuated using a set of neutral-density filters. Emitted light was collected from either the surface (for spontaneous PL) or from the cleaved facet (for SE) of the sample and analyzed by a Bruker Vertex 80v Fourier-transform spectrometer equipped with an MCT photodetector (16 µm cut-off wavelength) and operating in the step-scan mode. During SE studies, the excitation spot completely covered the entire sample, and each sample was placed in the center of the laser spot. Note that due to a specific growth direction (013), naturally cleaved facets did not form a Fabry–Perot resonator, so we predominantly studied single-pass SE. Additional SE experiments were conducted with another excitation source, an OPO emitting at 1.6 μm (also 10 ns pulse and a repetition rate of 10 Hz), to allow direct comparison with results from [20]. However, in this case only the “2.5 mm” sample could be effectively excited due to much lower pulse energy (~1 mJ). All experiments were conducted at room temperature with the samples mounted on a bulk aluminum heatsink.

The scattered 1.6 µm OPO (i) excitation and 808 nm diode excitation were completely cut off by a Ge filter limiting the spectrometer range to 550–5600 cm^−1^. However, we were unable to completely filter 2 µm OPO (ii) excitation from 2.7 µm SE, which greatly complicated spontaneous emission studies under pulsed excitation.

## 3. Results and Discussion

**Figure 2:**
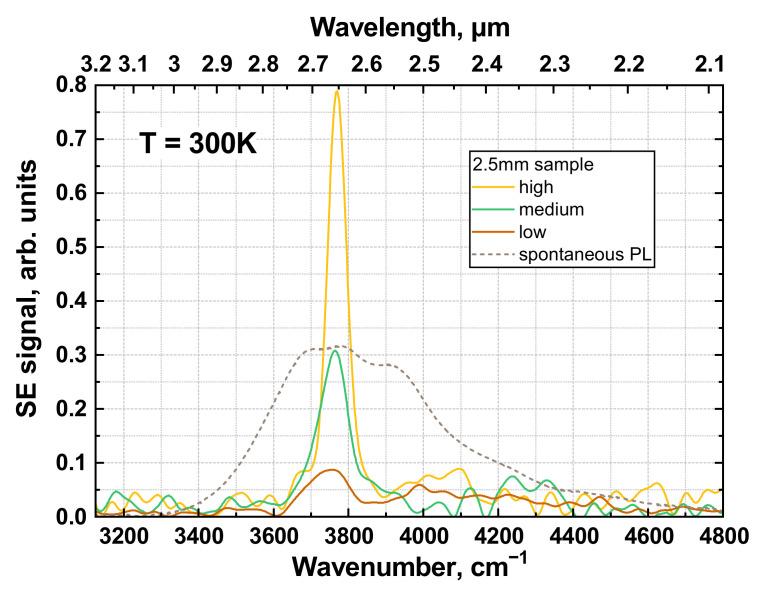
Stimulated emission (SE) spectra (solid lines) at 2.65 µm under various excitation intensities of 1.6 µm pulsed excitation (source (i)) and spontaneous emission spectrum under 808 nm continuous-wave diode excitation in the 2.5 mm sample. The low excitation intensity corresponded to less than 2 × 10^24^ s^−1^cm^−2^ photon flux, the medium one corresponded to ~(3 ÷ 3.5) × 10^24^ s^−1^cm^−2^ photon flux, and the high one to at least 4.5 × 10^24^ s^−1^cm^−2^ photon flux.

In Figure 2, we show a steady-state PL spectrum of the 2.5 mm sample under weak CW excitation alongside the emission spectra measured under pulsed 1.6 µm excitation (also used in our preceding work [20]). From the spontaneous PL, the band gap energy was estimated at *E*_g_~450 meV, which corresponds to ~18% Cd content in the QWs—close to the value retrieved from in situ sample characterization. Under pulsed excitation, a weak, broad, spontaneous emission spectrum could be observed at the lowest photon flux of 2 × 10^24^ s^−1^cm^−2^ with only a slight feature resembling the SE line, while, at higher pump densities, we observed rapid signal growth and spectrum narrowing down to ~60 cm^−1^ (for spontaneous PL, the full width at half maximum, FWHM, of the emission spectrum is ~500 cm^−1^), which marks the onset of stimulated emission. Note, again, that the excitation beam was focused into a ~3 mm spot to achieve sufficient pump intensity (≥200 kW/cm^2^) for SE generation, so we could only observe SE in the shortest 2.5 mm sample. The estimated SE threshold was about two times higher than the shorter-wavelength sample from [20], predominantly due to narrower E_g_ and, correspondingly, the lower Auger threshold energy E_th_ (~71 vs. ~65 meV).

**Figure 3:**
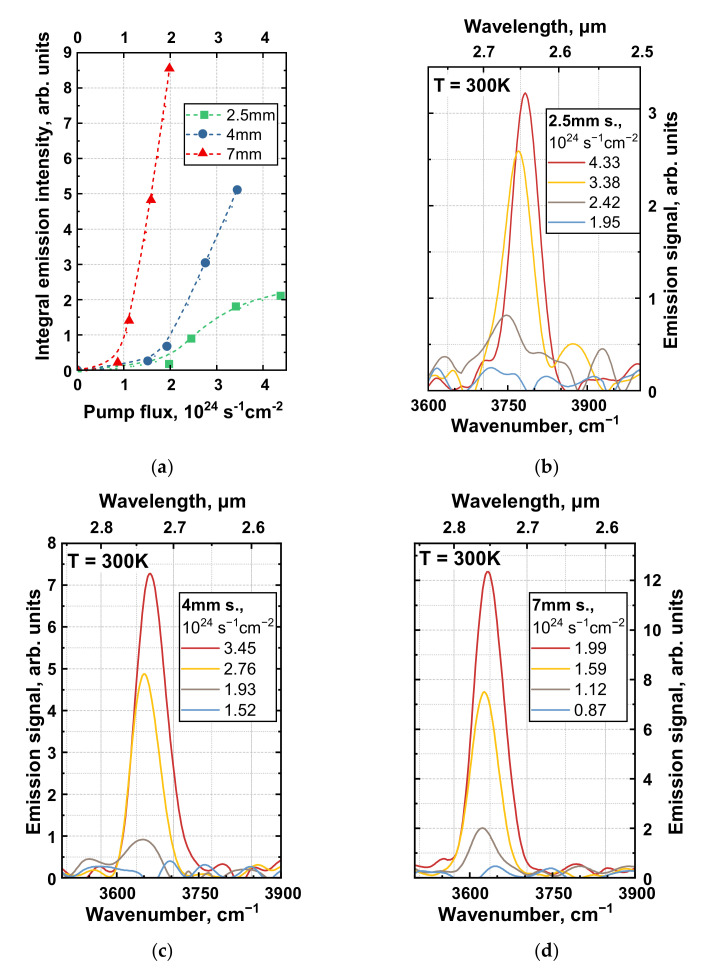
(**a**) Room-temperature light-in versus light-out (L–L curves) characteristics for each sample. Emission spectra of the (**b**) 2.5 mm sample, (**c**) 4 mm sample, (**d**) 7 mm sample under various excitation photon fluxes of the 2 µm pulsed excitation (source (ii)). Arbitrary units in (**b**–**d**) are the same for all three samples.

For more detailed studies, we switched to 2 µm OPO for the excitation of SE. With much higher pulse energy, it provided a photon flux high enough for SE generation in all samples and allowed accurate measurement of SE threshold intensities (see Figure 3). Note that, for the 2.5 mm sample, rather close SE thresholds were observed under 1.6 μm and 2 μm excitation at ~(2 ÷ 3) × 10^24^ s^−1^cm^−2^. While it was demonstrated in [20] that implementation of longer-wavelength excitation might lower the SE threshold, here, we did not see any pronounced effect. This can be explained given that the excitation wavelengths were both close to the SE wavelength (2.7 µm) and so the effects related to “hot-hole generation” (holes above the AR threshold, the concept proposed in [20]) and the strong heating of excess carriers at extreme pump levels (recently evaluated in [23]) were expected to be of minor importance. It should also be noted that both the 1.6 μm and 2 μm wavelengths corresponded to below-barrier excitation; based on measured transmittance spectra, we estimated direct absorption of the pump light in the QW array at some 8–10% at these wavelengths.

For each sample in the series, we measured SE spectra at several different excitation intensities determined by the set of optical filters. Experimental results are summarized in Figure 3. In panel (a), we show the integrated emission intensity depending on the excitation density; here, excitation photon fluxes for each sample were calculated, taking into account a Gaussian intensity profile of the pump laser beam (~6 mm FWHM) and the exact position of the sample relative to the excitation spot (shorter samples at the center of the laser spot effectively had higher photon flux than longer samples under the same average excitation power).

Clearly, luminescence had threshold dependence on the pump power, and samples of varying lengths exhibited different SE thresholds. Though there were minor variations in the band gap energies of the samples under study (see corresponding SE spectra in Figure 3b–d), it is not likely that interband recombination mechanisms and lasing thresholds were affected by this factor. Instead, we suggest that the observed differences in threshold intensities were directly related to the single-pass amplification regime, with longer samples allowing for a well-developed SE line at lower gain values. To provide rough estimates of material gain, we considered a one-dimensional amplifier model [24] and, based on the SE spectra given in Figure 3c,d, deduced gain values of ~60 cm^−1^ for the 7 mm sample at 1.6 × 10^24^ s^−1^cm^−2^ and ~110 cm^−1^ for the 4 mm sample at 2.75 × 10^24^ s^−1^cm^−2^ at the maximum of the SE line. Here, we accounted for an optical confinement factor of Γ~0.0355, identical for all samples.

Obviously, the 2.5 mm sample required even higher optical gain (and, hence, excitation intensity) to achieve distinct SE, and it was difficult to give any reliable estimates for this sample. The first problem here was the rather poor signal-to-noise ratio. Second, more importantly, we observed saturation of the emission intensity and blueshift of the SE line in Figure 3a,b above 3 × 10^24^ s^−1^cm^−2^, indicating carrier overheating at given pump densities. For the 2.5 mm sample, Figure 3a looked like the left part of a “bell-shaped” L–L curve, which we often observed in mid-IR HgCdTe QW heterostructures at high excitation intensity and at temperatures close to the critical temperature of SE generation [23,25]. This result was in a good agreement with positions of the SE peaks (and correspondinglythe maxima in the gain spectra were defined by the effective carrier temperature). Our previous experimental studies showed effective carrier temperature to be up to several times higher than the lattice temperature under the same 2 μm pumping as the excitation intensity increased beyond a certain value [23]. As the carrier temperature rises, the broadening of the gain spectrum triggers blueshift of the SE peak. The effect can hardly be avoided; however, its contribution can be minimized once dedicated photonic structures (e.g., Fabri–Perot cavities based on ridge waveguides) are fabricated. In this case, progressing from single-pass amplified spontaneous emission towards proper (multi-pass) lasing was expected to provide compact designs with a threshold intensity of less than 10^24^ s^−1^cm^−2^, corresponding to less than 1.5 kA/cm^2^ per QW under electrical pumping.

The given estimates for the threshold current of mid-IR HgCdTe lasers under consideration were definitely higher than those reported for III–V based lasers emitting at the same wavelength [26]. Still, HgCdTe may have certain possibilities for improvement. With a threshold flux at 10^24^ ph/(cm^2^ s) and a calculated QW transparency concentration of 10^12^ cm^−2^, we followed [23] to evaluate the effective (2D) Auger coefficient at 10^−14^ cm^4^/s, more than an order of magnitude higher than typically reported for III–V type-I QWs [26] and closely resembling “bulk” values. At the same time, QW-induced suppression of the Auger recombination at somewhat longer wavelengths was demonstrated in [18]. We suggest that the stronger AR in relatively wide gap samples studied in the current work might be related to the QW-specific processes with the excitation resulting in hot electrons in the continuum states above the barrier level (and it is exactly the process believed to be a limiting factor for high-temperature SE in [18]). While the energy threshold for conventional *eeh* AR (involving fundamental QW subbands) amounted to approximately 65 meV, we obtained about twice the lower value for barrier-assisted AR. Thus, tuning the latter process off-resonance by alternating barrier composition (and so the band offsets in the QW) may be helpful to mitigate related AR and reduce the lasing threshold to more practical levels.

## 4. Conclusions

We obtained stimulated emission at room temperature in the 2.65–2.75 µm range from a planar Hg_0.82_Cd_0.18_Te/Cd_0.6_Hg_0.4_Te QW structure under pulsed optical excitation. While this region is not very convenient for spectroscopy of gases in the atmosphere due to fundamental absorption lines of water and CO_2_, there are some potential applications for the sensing of these gases in the range discussed and in medicine. In comparison with previous structure design [20], the emission wavelength was 0.3 µm longer, while the threshold intensity for SE generation was several times higher under both 1.6 µm and 2 µm excitation. This required new structure designs for propagating the SE wavelength of room-temperature HgCdTe optical converters towards the 3–5 µm atmospheric window, in particular, active medium engineering for lowering the SE threshold intensity. Having studied a series of three samples with varying lengths, we observed pump-induced carrier heating while measuring signal vs. pump curves, and the effect was clearly pronounced in the shortest sample. We attribute this effect to the non-uniformity of the excitation beam profile and considered cavity processing to mitigate it.

## Data Availability

The data presented in this study are available on request from the corresponding author.
